# Analysis of bronchovascular patterns in the left superior division segment to explore the relationship between the descending bronchus and the artery crossing intersegmental planes

**DOI:** 10.3389/fonc.2023.1183227

**Published:** 2023-05-24

**Authors:** Zhikai Li, Qingtao Zhao, Wenbo Wu, Zhonghui Hu, Xiaopeng Zhang

**Affiliations:** ^1^ Graduate School, Hebei Medical University, Shijiazhuang, China; ^2^ Department of Thoracic Surgery, Hebei General Hospital, Shijiazhuang, China

**Keywords:** left superior division segment (LSDS), anatomical variation, artery crossing intersegmental planes, non-small cell lung carcinoma (NSCLC), segmentectomy, video-assisted thoracoscopic surgery (VATS)

## Abstract

**Background:**

A comprehensive understanding of the anatomical variations in the pulmonary bronchi and arteries is particularly essential to the implementation of safe and precise left superior division segment (LSDS) segmentectomy. However, no report shows the relationship between the descending bronchus and the artery crossing intersegmental planes. Thus, the purpose of the present study was to analyze the branching pattern of the pulmonary artery and bronchus in LSDS using three-dimensional computed tomography bronchography and angiography (3D-CTBA) and to explore the associated pulmonary anatomical features of the artery crossing intersegmental planes.

**Materials and methods:**

The 3D-CTBA images of 540 cases were retrospectively analyzed. We reviewed the anatomical variations of the LSDS bronchus and artery and assorted them according to different classifications.

**Results:**

Among all 540 cases of 3D-CTBA, there were 16 cases (44.4%) with lateral subsegmental artery crossing intersegmental planes (AX^3^a), 20 cases (55.6%) Without AX^3^a in the descending B^3^a or B^3^ type, and 53 cases (10.5%) with AX^3^a, 451 cases (89.5%) Without AX^3^a in the Without the descending B^3^a or B^3^ type. This illustrated that the AX^3^a was more common in the descending B^3^a or B^3^ type (P < 0.005). Similarly, there were 69 cases (36.1%) with horizontal subsegmental artery crossing intersegmental planes (AX^1 + 2^c), 122 cases (63.9%) Without AX^1 + 2^c in the descending B^1 + 2^c type, and 33 cases (9.5%) with AX^1 + 2^c, 316 cases (90.5%) Without AX^1 + 2^c in the Without the descending B^1 + 2^c type. Combinations of the branching patterns of the AX^1 + 2^c and the descending B^1 + 2^c type were significantly dependent (p < 0.005). The combinations of the branching patterns of the AX^1 + 2^c and the descending B^1 + 2^c type were frequently observed.

**Conclusions:**

This is the first report to explore the relationship between the descending bronchus and the artery crossing intersegmental planes. In patients with the descending B^3^a or B^3^ type, the incidence of the AX^3^a was increased. Similarly, the incidence of the AX^1 + 2^c was increased in patients with the descending B^1 + 2^c type. These findings should be carefully identified when performing an accurate LSDS segmentectomy.

## Introduction

With the widespread use of High-resolution computed tomography (HRCT), an increasing number of ground-glass opacities (GGOs) are being identified. Several studies have shown the same oncologic efficacy between a video-assisted thoracoscopic surgery (VATS) lobectomy and segmentectomy for GGO-dominant peripheral lung cancer (1–7).

The JCOG0804 study evaluates the efficacy and safety of sublobar resection for GGO-dominant peripheral lung cancer with consolidation tumor ratio ≤0.25 and maximum tumor diameter ≤2.0 cm (1). Based on the result of JCOG0804, sublobar resection with enough surgical margin offered sufficient local control and relapse-free survival (RFS) for GGO-dominant peripheral lung cancer (1). The JCOG0802 study is a randomized, controlled, non-inferiority trial to confirm whether segmentectomy is not inferior to lobectomy regarding prognosis (2, 3). The JCOG0802 study showed segmentectomy to be non-inferior and superior to lobectomy with regards to overall survival (OS) and concluded segmentectomy should be the standard surgical procedure, rather than lobectomy, for patients with small-sized (≤2 cm, consolidation-to-tumor ratio >0.5) peripheral non-small cell lung carcinoma (NSCLC) (2, 3).

The primary forms of sublobar resection currently contain wedge resection and anatomical segmentectomy. However, VATS segmentectomy is more complex than a standard lobectomy because of the anatomical sophistication of the lung, characterizing both segmental vessels and bronchi structures that diversify at different levels. Therefore, comprehensive knowledge of the pulmonary bronchovascular pattern by general thoracic surgeons has become more significant to the implementation of safe and precise left superior division segment (LSDS) surgery. However, only a few studies represent anatomic variations of the LSDS using three-dimensional computed tomography bronchography and angiographyy (3D-CTBA) (8–12). Moreover, we also found the aberrant artery crossing intersegmental planes in LSDS (13). The purpose of the present study was to classify the branching patterns of the left superior division bronchus (LSDB) and the left superior division artery (LSDA) by using data obtained from 3D-CTBA. Furthermore, we explore the associated anatomical features of artery crossing intersegmental planes in LSDS.

## Methods

### Patient preparation and reconstruction of 3D-CTBA

The inclusion and exclusion criteria of this study:

Inclusion criteria:

①. GGO, with a diameter of less than 2 cm and with a consolidation tumor ratio of less than 25%, situated in the left upper lobe (LUL);
②. Sublobar resection (segmentectomy or wedge resection) was implemented;
③. Patients underwent routine chest-enhanced CT examinations preoperatively.
④. Without a history of left lung surgery;

Exclusion criteria:

①. The images showed by enhanced CT lung examination were not distinct, which influenced the three-dimensional (3D) reconstruction of the lung;
②. The lesion size of the LUL exceeded 3cm.

From October 2020 to October 2022, 540 patients (248 men, 292 women; mean age, 56 years) were enrolled from the Department of Thoracic Surgery, Hebei General Hospital. After collecting CT data, the volume data from both arterial and venous phases were imported into a reconstruction software (Infer Operate Thorax Planning), which computed and processed the data before presenting it in 3D-CTBA images (14). All procedures involving human participants in this study were in accordance with the Declaration of Helsinki (revised in 2013). The Research Ethics Committee approved this retrospective study at Hebei General Hospital (no. 2022119). The need for patient consent was waived because of the retrospective nature of the study. Variations in the LSDA and LSDB were classified and summarized.

### Definition of each segment in the LUL

The LSDS and lingular segment (LS) form LUL. The LUL was sorted into the apicoposterior segment (S^1 + 2^), anterior segment (S^3^), superior lingular segment (S^4^), and inferior lingular segment (S^5^). S^1 + 2^ and S^3^ were subclassified into three pulmonary subsegments (S^1 + 2^a, S^1 + 2^b, S^1 + 2^c, S^3^a, S^3^b, S^3^c) respectively. The LSDS is consists of S^1 + 2^ and S^3^. The LS is comprised of S^4^ and S^5^.

### Definition of the LSDB and lingular segment bronchus (LSB)

**Figure 1:**
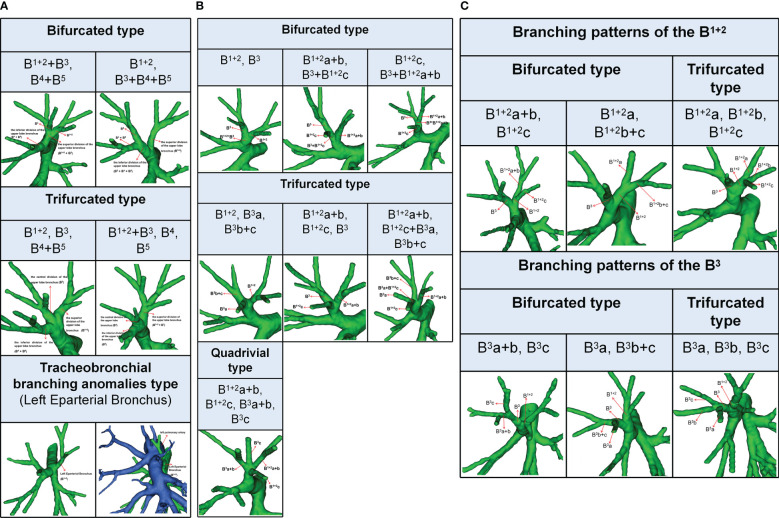
**(A)** 3D reconstruction model of branching patterns of the Left upper lobe bronchus.**(B)** 3D reconstruction model of branching patterns of the Left superior division bronchus (B^1 + 2^ + B^3^). **(C)** 3D reconstruction model of branching patterns of the B^1 + 2^ and B^3^.

Segmental and subsegmental bronchi of LSDB were nominated (10): B^1 + 2^ is the apicoposterior segmental bronchus that divides into apical (B^1 + 2^a), posterior (B^1 + 2^b) and horizontal ramus (B^1 + 2^c); B^3^ is the anterior segmental bronchus that is further sorted into lateral (B^3^a), medial (B^3^b) and superior ramus (B^3^c) (**Figure 1**). LSB divides into superior (B^4^) and inferior (B^5^) segmental bronchi (**Figure 1**).

According to the classification proposed by Dominique (15), the term left eparterial bronchus refers to any bronchus directed toward the LUL that originates from the left main bronchus (LMB) above the level where the left pulmonary artery (LPA) crosses the LMB (**Figure 1A**).

### Definition of the LSDA and the lingular segment artery (LSA)

**Figure 2:**
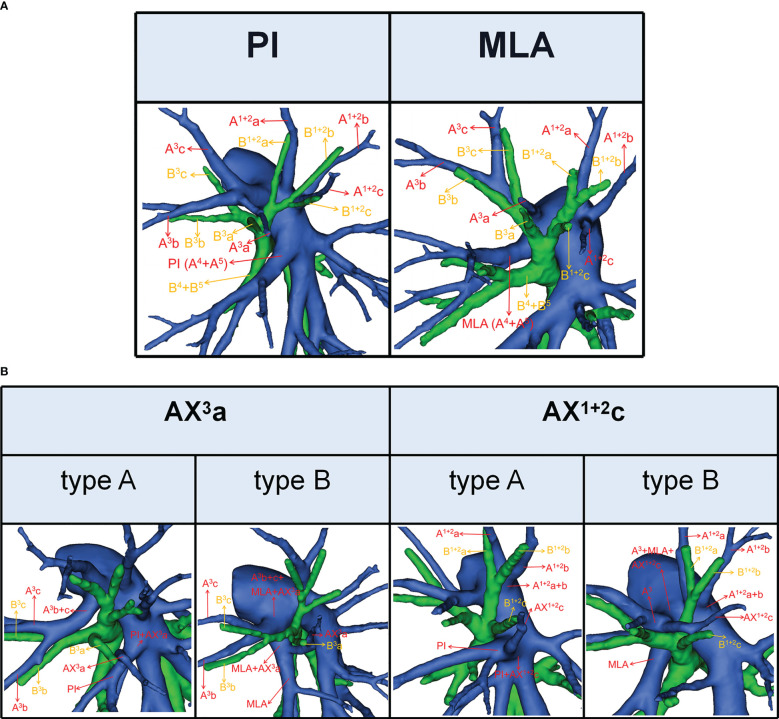
**(A)** 3D reconstruction model of PI and MLA. **(B)** 3D reconstruction model of AX^3^a: type A, AX^3^a originates from PI; type B, AX^3^a originates from MLA. 3D reconstruction model of AX^1 + 2^c: type A, AX^1 + 2^c originates from PI; type B, AX^1 + 2^c originates from A^3^.

Segmental and subsegmental arteries of LSDA were named (10): A^1 + 2^ is the apicoposterior segmental artery that divides into apical (A^1 + 2^a), posterior (A^1 + 2^b) and horizontal ramus (A^1 + 2^c); A^3^ is the anterior segmental artery that is further sorted into lateral (A^3^a), medial (A^3^b) and superior ramus (A^3^c) (**Figure 2A**). The LSA was comprised of the superior lingular artery (A^4^) and inferior lingular artery (A^5^).

According to the origin of LSA, the nomenclature of LSA was divided into two types (11): PI, which originates from the interlobar portion of the LPA; MLA, which originates from the mediastinal portion of the LPA (**Figure 2A**).

### Definition of the artery crossing intersegmental planes

The lateral subsegmental artery crossing intersegmental planes was defined as the AX^3^a (16). And AX^3^a has two origins (**Figure 2B**). When AX^3^a originates from PI, it spans the intersegmental planes between S^3^ and S^4^ and supplies S^3^a (**Figure 2B**). Similarly, when AX^3^a originates from MLA, it also spans the intersegmental planes between S^3^ and S^4^ and supplies S^3^a (**Figure 2B**).

The horizontal subsegmental artery crossing intersegmental planes was named as AX^1 + 2^c. Moreover, AX^1 + 2^c has two origins (**Figure 2B**). When AX^1 + 2^c originates from PI, it spans the intersegmental planes between S^3^ and S^4^ and supplies S^1 + 2^c (**Figure 2B**). However, when AX^1 + 2^c originates from A3, it spans the intersegmental planes between S^1 + 2^ and S^3^ and supplies S^1 + 2^c (**Figure 2B**).

### Statistics

All statistical analyses were implemented using SPSS 23.0 (SPSS, Chicago, IL, USA). Qualitative data were presented as the number of cases (percentage). The Pearson Chi-Square test was used to evaluate the significance of dependencies between the groups. A P-value less than 0.05 was considered statistically significant.

## Results

### Branching patterns of the LUL bronchus

The branching patterns of the LUL bronchus were classified into three types (**Table 1**; **Figure 1A**): bifurcated type (493/540, 91.3%), trifurcated type (45/540, 8.3%), and tracheobronchial branching abnormalities type (2/540, 0.4%). The bifurcated type was further divided into two subtypes (**Table 1**; **Figure 1A**): type 1, in which a common trunk of B^1 + 2^+B^3^ originates from the superior division of the upper lobe bronchus and a common trunk of B^4^+B^5^ originates from the inferior division of the upper lobe bronchus (90.9%); type 2, in which B^1 + 2^ originates from the superior division of the upper lobe bronchus and a common trunk of B^3^+B^4^+B^5^ originates from the inferior division of the upper lobe bronchus (0.4%). The trifurcated type was also further sorted into two subtypes (**Table 1**; **Figure 1A**): type 1, in which B^1 + 2^ comes from the superior division of the upper lobe bronchus, B^3^ comes from the central division of the upper lobe bronchus, and a common trunk of B^4^+B^5^ comes from the inferior division of the upper lobe bronchus (3.9%); type 2, in which a common trunk of B^1 + 2^+B^3^ comes from the superior division of the upper lobe bronchus, B^4^ comes from the central division of the upper lobe bronchus, and B^5^ comes from the inferior division of the upper lobe bronchus (4.4%). Moreover, B^1 + 2^ originating from the LMB was found in 2 cases in the Left Eparterial Bronchus. The LPA was on the ventral side of B^1 + 2^ and did not cross the dorsal side of the LMB (**Figure 1A**).

**Table 1:** Branching patterns of the Left upper lobe bronchus.

	Our study (n=540)		Maki (n=539)		Deng (n=103)		Wang (n=166)	
	NO.	%	NO.	%	NO.	%	NO.	%
Bifurcated type	493	91.3	528	98.0	92	89.3	165	99.4
B^1 + 2^+B^3^, B^4^+B^5^	491	90.9	528	98.0	92	89.3	164	98.8
B^1 + 2^, B^3^+B^4^+B^5^	2	0.4	NR	–	NR	–	1	0.6
Trifurcated type	45	8.3	9	1.7	11	10.7	1	0.6
B^1 + 2^, B^3^, B^4^+B^5^	21	3.9	9	1.7	11	10.7	1	0.6
B^1 + 2^+B^3^, B^4^, B^5^	24	4.4	NR	–	NR	–	NR	–
Tracheobronchial branching anomalies type	2	0.4	2	0.3	NR	–	NR	–
Left Eparterial Bronchus	2	0.4	2	0.3	NR	–	NR	–

NR: the type was not referred

### Branching patterns of the LSDB (B^1 + 2^ + B^3^)

The branching patterns of LSDB were divided into three types (**Table 2**; **Figure 1B**): bifurcated type (305/491, 62.1%), trifurcated type (185/491, 37.7%), and quadrivial type (1/491, 0.2%). The bifurcated type was further separated into three subtypes (**Table 2**; **Figure 1B**): subtype I (B^1 + 2^, B^3^), subtype II (B^1 + 2^a+b, B^3^+B^1 + 2^c), subtype III (B^1 + 2^c, B^3^+B^1 + 2^a+b). These subtypes accounted for 58.7%, 2.0%, and 1.4%, respectively. The trifurcated type was also further subclassified into three subtypes: subtype I (B^1 + 2^, B^3^a, B^3^b+c) was observed in 2.2% of cases, subtype II (B^1 + 2^a+b, B^1 + 2^c, B^3^) was the most common (35.0%) and subtype III (B^1 + 2^a+b, B^1 + 2^c+B^3^a, B^3^b+c) was seen in 2 cases (0.4%). The quadrivial type (B^1 + 2^a+b, B^1 + 2^c, B^3^a+b, B^3^c) was the less common (0.2%).

**Table 2:** Branching patterns of the Left superior division bronchus (B^1 + 2^ + B^3^).

	Our study (n=491)		Maki (n=537)		Deng (n=92)		Wang (n=166)	
	NO.	%	NO.	%	NO.	%	NO.	%
Bifurcated type	305	62.1	408	76.0	92	100.0	109	65.7
B^1 + 2^, B^3^	288	58.7	408	76.0	92	100.0	102	61.4
B^1 + 2^a+b, B^3^+B^1 + 2^c	10	2.0	NR	–	NR	–	6	3.6
B^1 + 2^c, B^3^+B^1 + 2^a+b	7	1.4	NR	–	NR	–	NR	–
B^1 + 2^+B^3^c, B^3^a+b	NR	–	NR	–	NR	–	1	0.6
Trifurcated type	185	37.7	129	24.0	NR	–	57	34.3
B^1 + 2^, B^3^a, B^3^b+c	11	2.2	18	3.4	NR	–	8	4.8
B^1 + 2^a+b, B^1 + 2^c, B^3^	172	35.0	109	20.3	NR	–	48	28.9
B^1 + 2^a+b, B^1 + 2^c+B^3^a, B^3^b+c	2	0.4	NR	–	NR	–	NR	–
B^1 + 2^a, B^1 + 2^b+c, B^3^	NR	–	NR	–	NR	–	1	0.6
B^1 + 2^, B^3^a+b, B^3^c	NR	–	2	0.3	NR	–	NR	–
Quadrivial type	1	0.2	NR	–	NR	–	NR	–
B^1 + 2^a+b, B^1 + 2^c, B^3^a+b, B^3^c	1	0.2	NR	–	NR	–	NR	–

NR: the type was not referred

### Branching patterns of the B^1 + 2^, B^3^

The branching pattern of B^1 + 2^ included two types: 522 cases were bifurcated, while trifurcated was found only in 18 cases (**Table 3**; **Figure 1C**). The bifurcated type was further divided into subtype I (B^1 + 2^a+b, B^1 + 2^c), which was presented in 488 patients (90.4%) and subtype II (B^1 + 2^a, B^1 + 2^b+c), that was occurred in 34 patients (6.3%) (**Figure 1C**). Similarly, the branching pattern of B^3^ contained two types: bifurcated type (445/540, 82.4%) and trifurcated type (95/540, 17.6%) (**Table 3**; **Figure 1C**). The bifurcated type was further divided into subtype I (B^3^a+b, B^3^c), which was found in 70 patients (13.0%) and subtype II (B^3^a, B^3^b+c), which was the most common (69.4%) (**Figure 1C**).

**Table 3:** Branching patterns of the B^1 + 2^ and Branching patterns of the B^3^.

	Our study (n=540)		Wang (n=166)	
	NO.	%	NO.	%
Branching patterns of the B^1 + 2^				
Bifurcated type	522	96.7	166	100.0
B^1 + 2^a+b, B^1 + 2^c	488	90.4	156	94.0
B^1 + 2^a, B^1 + 2^b+c	34	6.3	10	6.0
Trifurcated type	18	3.3	NR	–
B^1 + 2^a, B^1 + 2^b, B^1 + 2^c	18	3.3	NR	–
Branching patterns of the B^3^				
Bifurcated type	445	82.4	157	94.6
B^3^a+b, B^3^c	70	13.0	19	11.4
B^3^a, B^3^b+c	375	69.4	138	83.1
Trifurcated type	95	17.6	9	5.4
B^3^a, B^3^b, B^3^c	95	17.6	9	5.4

NR: the type was not referred

### Branching patterns of the A^3^

**Figure 3:**
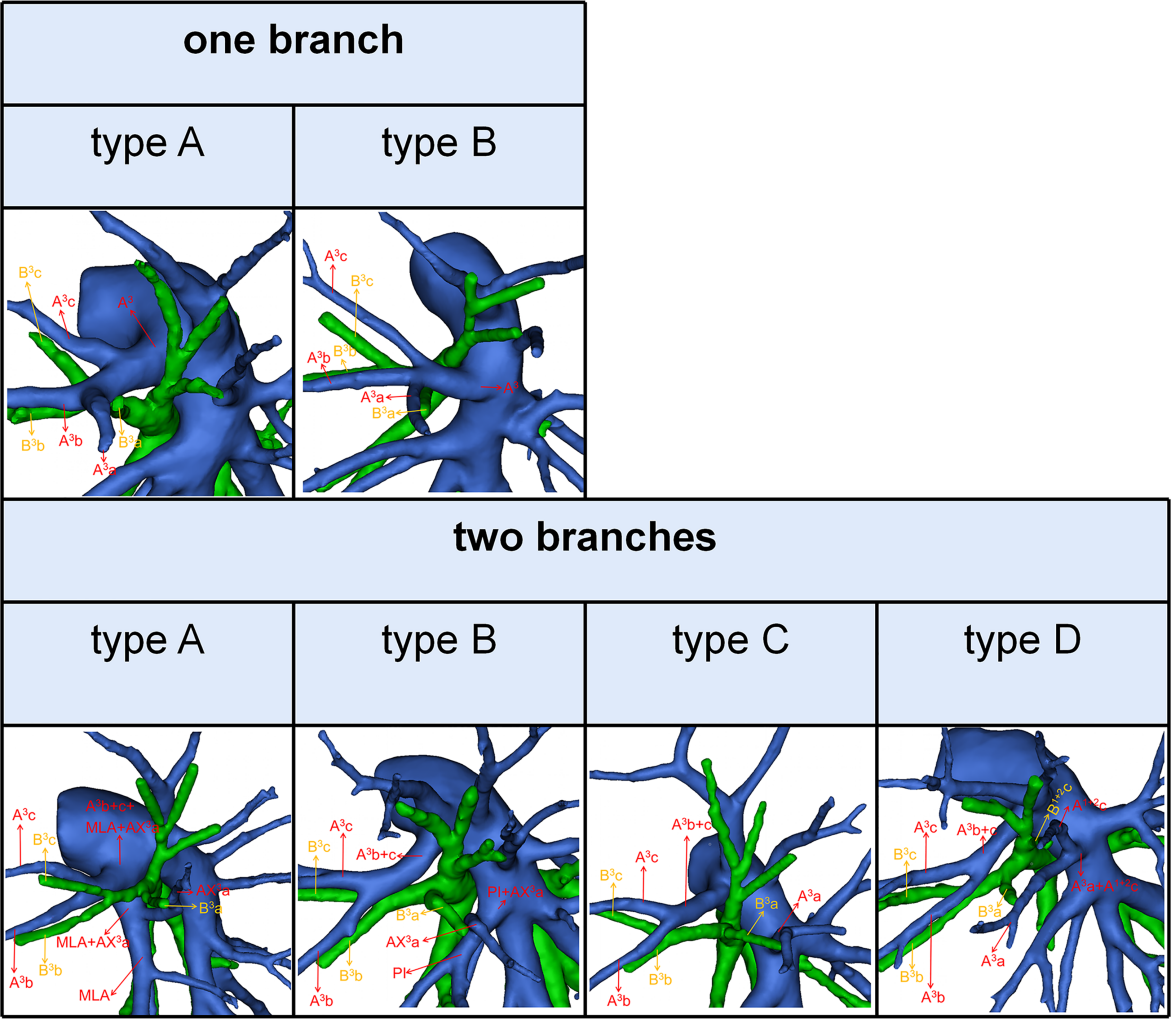
3D reconstruction model of branching patterns of the A^3^.

According to the original location and the number of the A^3^, the branching patterns of the A^3^ were classified and summarized in detail (**Table 4**; **Figure 3**). When the composition of the A^3^ included a single branch, the branching patterns of the A^3^ were classified into two types: type A, A^3^ from the anterior portion of LPA (83.0%); type B, A^3^ from the interlobar portion of LPA (0.5%). When the composition of the A^3^ contained two branches, the branching patterns of the A^3^ were divided into four types: type A, A^3^b+c from the anterior portion of LPA and AX^3^a from MLA (4.6%); type B, A^3^b+c from the anterior portion of LPA and AX^3^a from PI (8.1%); type C, A^3^b+c from the anterior portion of LPA and A^3^a from the interlobar portion of LPA (Independent A^3^a) (3.0%); type D, A^3^b+c from the anterior portion of LPA and A^3^a from the interlobar portion of LPA (A^3^a and A^1 + 2^c share a common trunk) (0.7%). As detailed in **Table 4**, the incidence of AX^3^a was 12.8% (69/540). Furthermore, we summarized the distribution of AX^3^a in bronchus type (**Table 5**).

**Table 4:** Branching patterns of the A^3^.

	Our study (n=540)	
	NO.	%
one branch	451	83.5
Type A (A^3^ from anterior portion of LPA)	448	83.0
Type B (A^3^ from interlobar portion of LPA)	3	0.5
two branches	89	16.5
Type A (A^3^b+c from anterior portion of LPA and AX^3^a from MLA)	25	4.6
Type B (A^3^b+c from anterior portion of LPA and AX^3^a from PI)	44	8.1
Type C (A^3^b+c from anterior portion of LPA and A^3^a from interlobar portion of LPA) (Independent A^3^a)	16	3.0
Type D (A^3^b+c from anterior portion of LPA and A^3^a from interlobar portion of LPA) (A^3^a and A^1 + 2^c share a common trunk)	4	0.7

**Table 5:** Distribution of the AX^3^a in bronchus type.

	With AX^3^a	Without AX^3^a	total
Bronchus type			
B^1 + 2^+B^3^, B^4^+B^5^			
B^1 + 2^, B^3^	48	240	288
B^1 + 2^a+b, B^3^+B^1 + 2^c	0	10	10
B^1 + 2^c, B^3^+B^1 + 2^a+b	0	7	7
B^1 + 2^, B^3^a, B^3^b+c	7	4	11
B^1 + 2^a+b, B^1 + 2^c, B^3^	5	167	172
B^1 + 2^a+b, B^1 + 2^c+B^3^a, B^3^b+c	1	1	2
B^1 + 2^a+b, B^1 + 2^c, B^3^a, B^3^b+c	0	1	1
B^1 + 2^, B^3^+B^4^+B^5^	0	2	2
B^1 + 2^, B^3^, B^4^+B^5^	8	13	21
B^1 + 2^+B^3^, B^4^, B^5^	0	24	24
Left Eparterial Bronchus	0	2	2
total	69	471	540

### Combinations of branching patterns of AX^3^a and the descending B^3^a or B^3^ type

**Figure 4:**
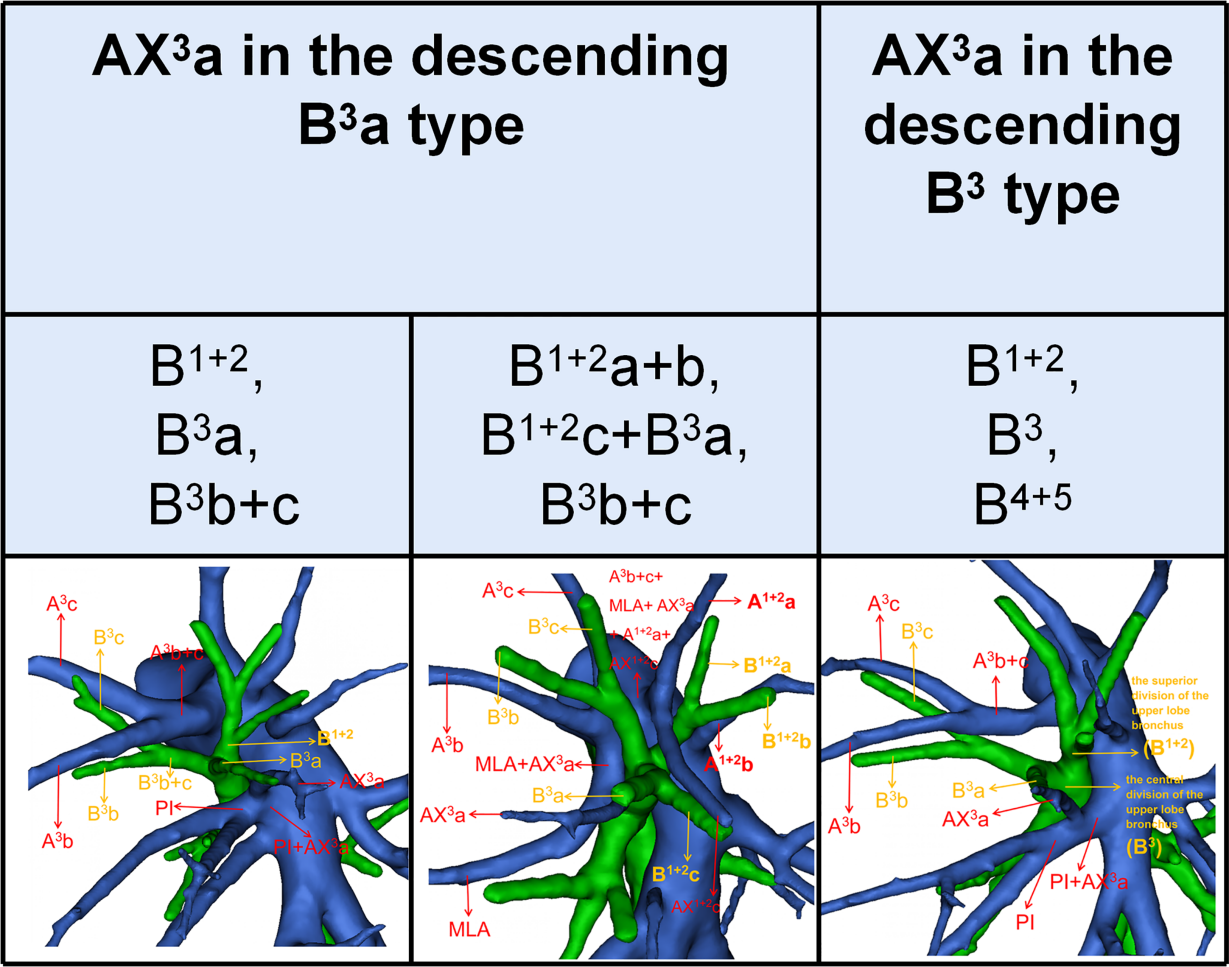
Distribution of the AX^3^a in the descending B^3^a or B^3^ type.

In the following types (**Table 2**; **Figure 1B**), B^1 + 2^, B^3^a, B^3^b+c (2.2%) and B^1 + 2^a+b, B^1 + 2^c+B^3^a, B^3^b+c (0.4%), the components of B^3^ comprised the descending B^3^a. Similarly, in the following types (**Table 1**; **Figure 1A**), B^1 + 2^, B^3^, B^4^+B^5^ (3.9%) and B^1 + 2^, B^3^+B^4^+B^5^ (0.4%), the components of B^3^ comprised the descending B^3^. As shown in **Figure 4** and **Table 6**, the incidence of AX^3^a with and without the descending B^3^a or B^3^ type was 44.4% (16/36) and 10.5% (53/504), respectively. This indicated that the AX^3^a was more common in the descending B^3^a or B^3^ type (P < 0.005).

**Table 6:** Distribution of the AX^3^a in the descending B^3^a or B^3^ type.

	With AX^3^a	Without AX^3^a	total	P value
With the descending B^3^a or B^3^	16	20	36	p < 0.005
Without the descending B^3^a or B^3^	53	451	504	
total	69	471	540	

### Branching patterns of the A^1 + 2^

**Figure 5:**
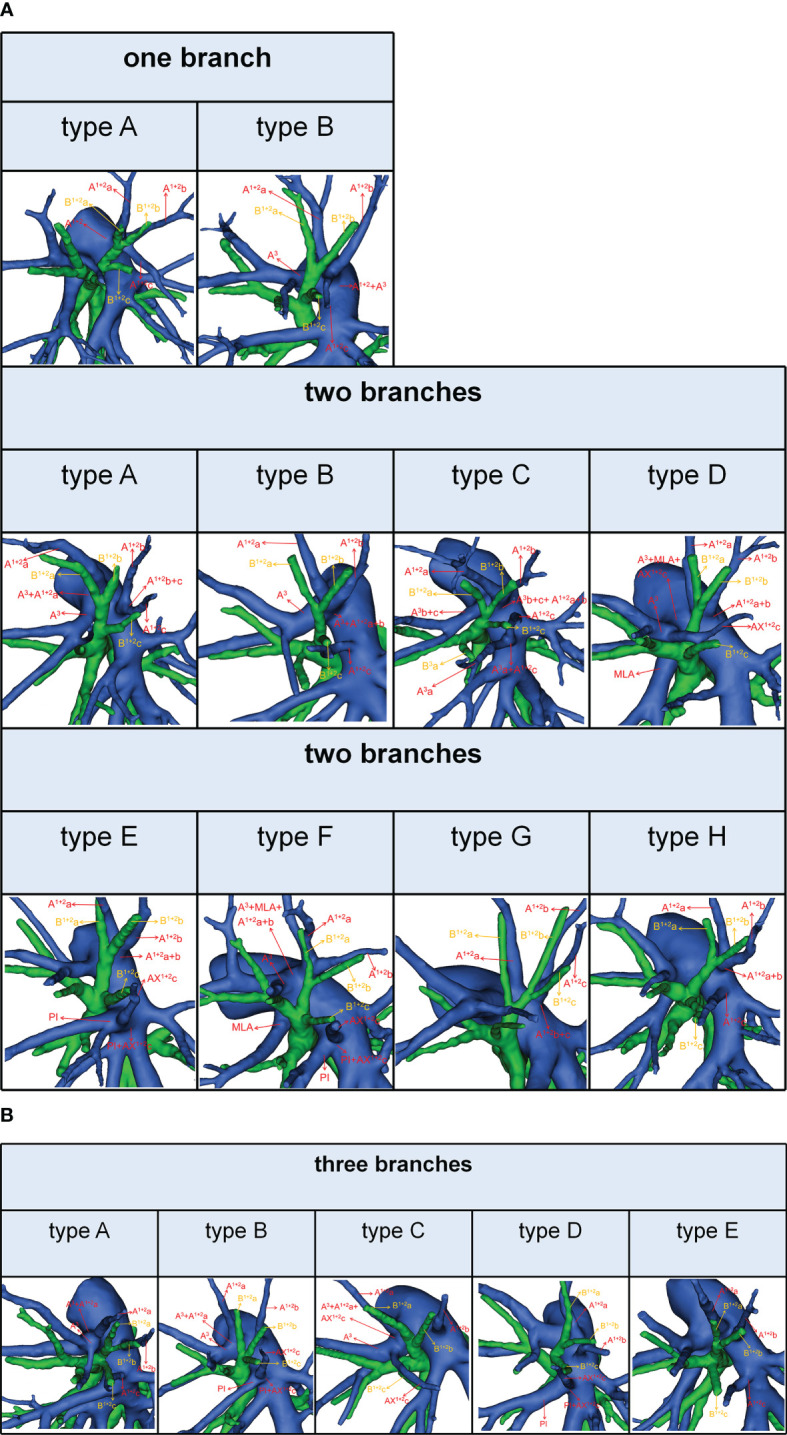
**(A)** 3D reconstruction model of branching patterns of the A^1 + 2^, when the composition of the A^1 + 2^ contained single branch and two branches. **(B)** 3D reconstruction model of branching patterns of the A^1 + 2^, when the composition of the A^1 + 2^ contained three branches.

According to the original location and the number of the A^1 + 2^, the branching patterns of the A^1 + 2^ were also sorted and summarized in detail (**Table 7**; **Figure 5**). When the composition of the A^1 + 2^ consisted of a single branch (**Figure 5A**), two types were defined: type A, A^1 + 2^ from the posterolateral portion of LPA (7.4%); type B, A^1 + 2^ from A^3^ (0.7%). When the composition of the A^1 + 2^ involves two branches, patients can be divided into one of the following eight types (**Figure 5A**): type A, A^1 + 2^a from A^3^ and A^1 + 2^b+c from posterolateral portion of LPA (13.9%); type B, A^1 + 2^a+b from A^3^ and A^1 + 2^c from interlobar portion of LPA (12.4%); type C, A^1 + 2^a+b from A^3^ and A^1 + 2^c from interlobar portion of LPA (A^3^a and A^1 + 2^c share a common trunk) (0.7%); type D, A^1 + 2^a+b from posterolateral portion of LPA and AX^1 + 2^c from A^3^ (2.4%); type E, A^1 + 2^a+b from posterolateral portion of LPA and AX^1 + 2^c from PI (2.4%); type F, A^1 + 2^a+b from A^3^ and AX^1 + 2^c from PI (1.7%); type G, A^1 + 2^a from posterolateral portion of LPA and A^1 + 2^b+c from posterolateral portion of LPA (3.0%); type H, A^1 + 2^a+b from posterolateral portion of LPA and A^1 + 2^c from interlobar portion of LPA (16.5%). When the composition of the A^1 + 2^ contained three branches, there are five types (**Figure 5B**): type A, A^1 + 2^a from A^3^, A^1 + 2^b from posterolateral portion of LPA and A^1 + 2^c from interlobar portion of LPA (22.4%); type B, A^1 + 2^a from A^3^, A^1 + 2^b from posterolateral portion of LPA and AX^1 + 2^c from PI (7.4%); type C, A^1 + 2^a from A^3^, A^1 + 2^b from posterolateral portion of LPA and AX^1 + 2^c from A3 (2.8%); type D, A^1 + 2^a from posterolateral portion of LPA, A^1 + 2^b from posterolateral portion of LPA and AX^1 + 2^c from PI (2.2%); type E, A^1 + 2^a from posterolateral portion of LPA, A^1 + 2^b from posterolateral portion of LPA and A^1 + 2^c from interlobar portion of LPA (4.1%). As shown in **Table 7**, the incidence of AX^1 + 2^c was 18.9% (102/540). Furthermore, we summarized the distribution of AX^1 + 2^c in bronchus type (**Table 8**).

**Table 7:** Branching patterns of the A^1 + 2^.

	Our study(n=540)		Chen (n=404)	
	NO.	%	NO.	%
one branch	44	8.1	53	13.1
Type A (A^1 + 2^ from the posterolateral portion of LPA)	40	7.4	53	13.1
Type B (A^1 + 2^ from A^3^)	4	0.7	NR	–
two branches	286	53.0	254	62.9
Type A (A^1 + 2^a from A^3^ and A^1 + 2^b+c from posterolateral portion of LPA)	75	13.9	68	16.8
Type B (A^1 + 2^a+b from A^3^ and A^1 + 2^c from interlobar portion of LPA)	67	12.4	49	12.1
Type C (A^1 + 2^a+b from A^3^ and A^1 + 2^c from interlobar portion of LPA) (A^3^a and A^1 + 2^c share a common trunk)	4	0.7	NR	–
Type D (A^1 + 2^a+b from posterolateral portion of LPA and AX^1 + 2^c from A^3^)	13	2.4	NR	–
Type E (A^1 + 2^a+b from posterolateral portion of LPA and AX^1 + 2^c from PI)	13	2.4	NR	–
Type F (A^1 + 2^a+b from A^3^ and AX^1 + 2^c from PI)	9	1.7	NR	–
Type G (A^1 + 2^a from posterolateral portion of LPA and A^1 + 2^b+c from posterolateral portion of LPA)	16	3.0	28	6.9
Type H (A^1 + 2^a+b from posterolateral portion of LPA and A^1 + 2^c from interlobar portion of LPA)	89	16.5	109	27.0
three branches	210	38.9	97	24.0
Type A (A^1 + 2^a from A^3^, A^1 + 2^b from posterolateral portion of LPA, and A^1 + 2^c from interlobar portion of LPA)	121	22.4	66	16.3
Type B (A^1 + 2^a from A^3^, A^1 + 2^b from posterolateral portion of LPA, and AX^1 + 2^c from PI)	40	7.4	NR	–
Type C (A^1 + 2^a from A^3^, A^1 + 2^b from posterolateral portion of LPA, and AX^1 + 2^c from A3)	15	2.8	NR	–
Type D (A^1 + 2^a from posterolateral portion of LPA, A^1 + 2^b from posterolateral portion of LPA, and AX^1 + 2^c from PI)	12	2.2	NR	–
Type E (A^1 + 2^a from posterolateral portion of LPA, A^1 + 2^b from posterolateral portion of LPA, and A^1 + 2^c from interlobar portion of LPA)	22	4.1	31	7.7

**Table 8:** Distribution of the AX^1 + 2^c in bronchus type.

	With AX^1 + 2^c	Without AX^1 + 2^c	total
Bronchus type			
B^1 + 2^+B^3^, B^4^+B^5^			
B^1 + 2^, B^3^	29	259	288
B^1 + 2^a+b, B^3^+B^1 + 2^c	5	5	10
B^1 + 2^c, B^3^+B^1 + 2^a+b	2	5	7
B^1 + 2^, B^3^a, B^3^b+c	0	11	11
B^1 + 2^a+b, B^1 + 2^c, B^3^	61	111	172
B^1 + 2^a+b, B^1 + 2^c+B^3^a, B^3^b+c	1	1	2
B^1 + 2^a+b, B^1 + 2^c, B^3^a, B^3^b+c	1	0	1
B^1 + 2^, B^3^+B^4^+B^5^	0	2	2
B^1 + 2^, B^3^, B^4^+B^5^	0	21	21
B^1 + 2^+B^3^, B^4^, B^5^	3	21	24
Left Eparterial Bronchus	0	2	2
total	102	438	540

### Combinations of branching patterns of AX^1 + 2^c and the descending B^1 + 2^c type

**Figure 6:**
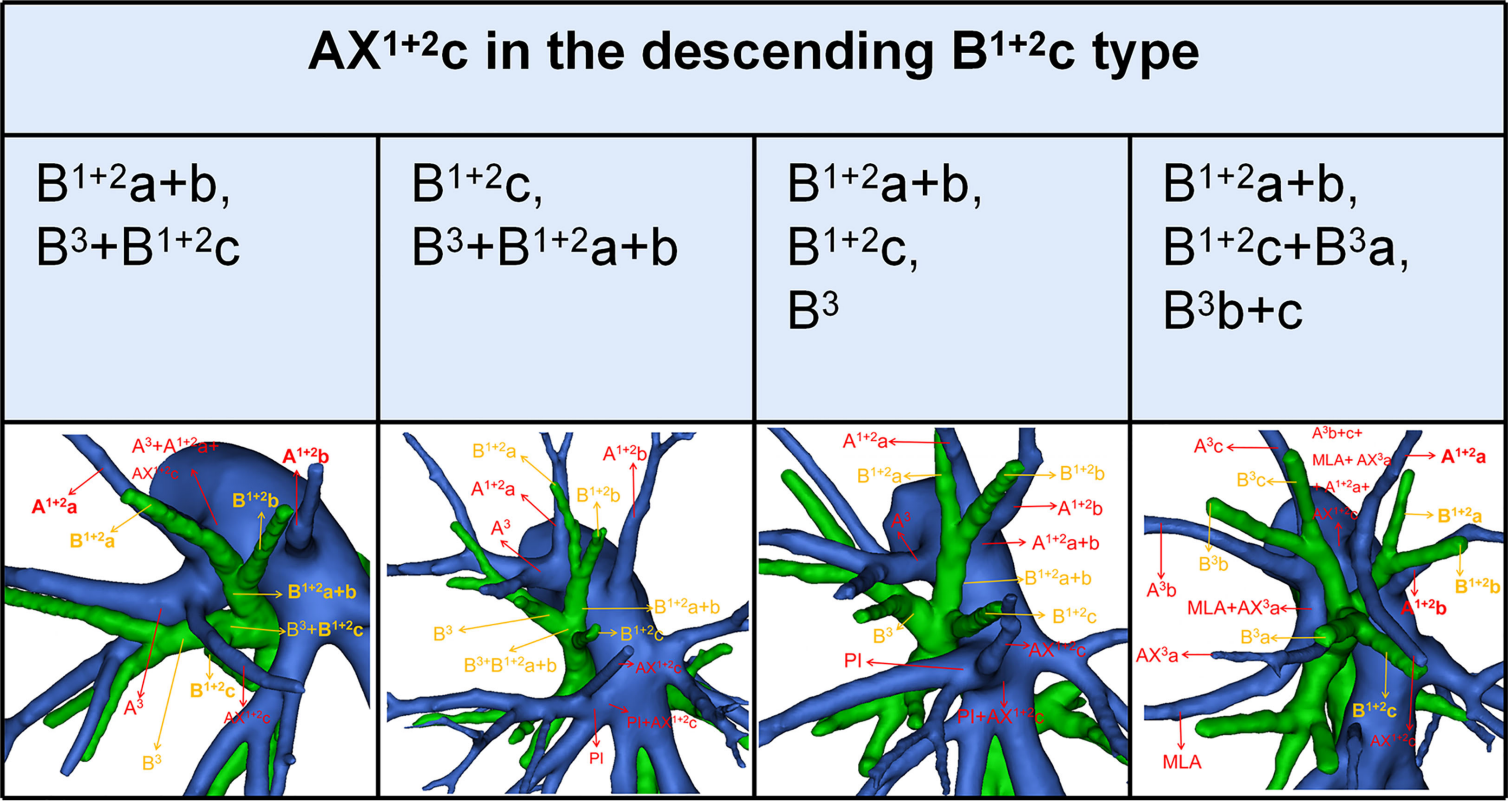
Distribution of the AX^1 + 2^c in the descending B^1 + 2^c type.

In the following types (**Table 2**; **Figure 1B**), B^1 + 2^a+b, B^3^+B^1 + 2^c (2.0%), B^1 + 2^c, B^3^+B^1 + 2^a+b (1.4%), B^1 + 2^a+b, B^1 + 2^c, B^3^ (35.0%), and B^1 + 2^a+b, B^1 + 2^c+B^3^a, B^3^b+c (0.4%), the components of B^1 + 2^ included the descending B^1 + 2^c. As shown in **Figure 6** and **Table 9**, the incidence of AX^1 + 2^c with and without the descending B^1 + 2^c type was 36.1% (69/191) and 9.5% (33/349), respectively. Combinations of the branching patterns of the AX^1 + 2^c and the descending B^1 + 2^c type were significantly dependent (p < 0.005). This indicates that the incidence of AX^1 + 2^c was increased in patients with the descending B^1 + 2^c type.

**Table 9:** Distribution of the AX^1 + 2^c in the descending B^1 + 2^c type.

	With AX^1 + 2^c	Without AX^1 + 2^c	total	P value
With the descending B^1 + 2^c	69	122	191	p < 0.005
Without the descending B^1 + 2^c	33	316	349	
total	102	438	540	

## Discussion

The low-dose thin-slice chest CT has extremely boosted the screening rate of GGOs. Some previous studies indicated that sublobar resection should be the standard surgical procedure for patients with GGO-dominant peripheral NSCLC (1–7). Identification and dissection of the branch of LSDA are significant parts of the LSDS anatomic segmentectomy. The anatomical variations of the branching pattern in the LSDA are diverse, which extraordinarily augments the challenge in the VATS segmentectomy of the LSDS. Intraoperative misunderstandings of the branch of LSDA can result in serious complications, such as uncontrollable intraoperative bleeding that convert to open thoracotomy. Moreover, pulmonary arterial injury, particularly the LUL, was a common source of hemorrhage (17). Therefore, thoracic surgeons must have a systemic and precise understanding of the branching pattern in the LSDA.

Fortunately, advances in HRCT and reconstruction techniques have allowed for the visualization of the branching pattern of the bronchus and vascular of the lungs (18, 19). The efficacy of blood vessel visualization is reportedly about 95.2% (20). The literature illustrated that 3D-CTBA is a valuable tool for thoracic surgeons to implement segmentectomy or subsegmentectomy procedures, which decreases operative-related complications and operation time and guarantees safe surgical margins (19).

However, there are only a few reports that have comprehensively summarized and sorted the branching pattern of LSDA and LSDB using 3D-CTBA (8–13). And the correlation of the descending bronchus and the artery crossing intersegmental planes in LSDS has not been reported in previous studies. In the present study, we detailedly analyzed the branching pattern of LSDA and LSDB and highlighted the differences between our results and those of previous reports (8–13). Furthermore, we explored the associated anatomical features of AX^3^a and AX^1 + 2^c.

In the present study, the branching patterns of the LUL bronchus were divided into three types (**Table 1**; **Figure 1A**). A common trunk of B^3^+B^4^+B^5^ originating from the inferior division of the upper lobe bronchus was a rare anomaly (0.4%), which was similar to the findings of Wang (0.6%) and Zhang (0.1%) (10, 21). When B^3^ originated from the B^4^+B^5^, the literature reported that it is often accompanied by the following two other variants: A, A^3^ comes from A^4^+A^5^ and V^1 + 2^c drained into the inferior pulmonary vein; B, additional fissure dividing the LUL into S^1 + 2^ and S^3 + 4+5^ (21). It is significant to note these malformations before the S^1 + 2^ segmentectomy is performed. Moreover, we found that the trifurcated type included two branching types (**Table 1**): B^1 + 2^, B^3^, B^4^+B^5^ type (3.9%), which incidence was higher than that of Maki (1.7%) and B^1 + 2^+B^3^, B^4^, B^5^ type, which has not been reported in the previous studies. When B^3^ originated the central division of the upper lobe bronchus, it is greatly easy to mistake the superior division of the upper lobe bronchus (B^1 + 2^) for B^1 + 2^+B^3^ during LSDS segmentectomy, which resulted in inadequate surgical safety margins. The Left Eparterial Bronchus was seen in 2 cases (0.4%), which incidence was similar to that of Maki (0.3%) (8). Previous reports demonstrated that the Left Eparterial Bronchus type has the following characteristics (1): the LPA does not cross the dorsal side of the displaced bronchus (2); incomplete lobulation exists between the LUL and left lower lobes (LLL) (22, 23). Therefore, whenever abnormalities of the pulmonary artery and incomplete lobulation are identified, it is crucial to keep an eye on the existence of the Left Eparterial Bronchus.

We found that the branching patterns of the LSDB had three types (**Table 2**; **Figure 1B**): bifurcated type (62.1%), which incidence was lower than that of Maki (76.0%) and Wang (65.7%); trifurcated type (37.7%), which was considerably higher than the frequency reported by Maki (24.0%) and Wang (34.3%); quadrivial type (0.2%), which was not found by Maki and Wang (8–10). In the bifurcated type, the B^1 + 2^, B^3^ type was the most common type (58.7%) and the B^1 + 2^c, B^3^+B^1 + 2^a+b type was first reported (8–10). However, we have not found the B^1 + 2^+B^3^c, B^3^a+b type (10). For the B^1 + 2^a+b, B^3^+B^1 + 2^c type, a mistaken ligation of the trunk of B^3^+B^1 + 2^c will result in lung volume loss in the S^3^ segmentectomy (**Figure 1B**). In trifurcated type, the B^1 + 2^a+b, B^1 + 2^c, B^3^ type was the most common type (35.0%), and the B^1 + 2^a+b, B^1 + 2^c+B^3^a, B^3^b+c type was not found in the previous literature. For the B^1 + 2^a+b, B^1 + 2^c+B^3^a, B^3^b+c type, a mistaken ligation of the trunk of B^1 + 2^c+B^3^a will lead to the enlarged intersegmental plane in the S^1 + 2^c segmentectomy (**Figure 1B**). Moreover, the B^1 + 2^a, B^1 + 2^b+c, B^3^ type and B^1 + 2^, B^3^a+b, B^3^c type were not detected in our study (8, 10).

We also observed that the branching patterns of the B^1 + 2^ had two types (**Table 3**; **Figure 1C**): the bifurcated type(96.7%), which incidence was similar to that of Wang (100.0%), and the trifurcated type (3.3%), which has not been reported in the literature (10). Moreover, the classification of the branching patterns of the B^3^ was the same as that of Wang (**Table 3**).

To our knowledge, the detailed classification of branching patterns of the A^3^ was first reported (**Table 4**; **Figure 3**). An understanding of the origin of the A^3^ branch is essential in clinical practice if a safe and precise S^3^ segmentectomy is to be implemented. When A^3^ originates from the interlobar portion of LPA, A^3^ can usually be identified by dissecting interlobar fissures. When AX^3^a arose from PI, AX^3^a should be carefully dissected from PI before it is ligated to avoid injuring PI. When a common trunk of A^1 + 2^c and A^3^a directly originated from the interlobar portion of the LPA, A^3^a should be resected without resecting A^1 + 2^c.

In the case of S^3^a segmentectomy, it is necessary to investigate the origin of A^3^a. In the present study, A^3^a arose from the interlobar portion of LPA in 20 cases (3.7%) (**Table 4**; **Figure 3**). This compares with the figures showed by Maki (3.9%) and Murota (8.4%) (8, 11). AX^3^a originating from the PI was observed in cases (8.1%), which incidence was similar to that of Maki (6.1%) and Murota (8.1%) (8, 11). Moreover, AX^3^a originating from MLA occurred in 25 patients (4.6%) and was the first reported (**Figure 2B**).

In our study, an interesting finding concerned the AX^3^a (**Table 5**; **Figure 4**). There was a significant correlation between the branching patterns of the AX^3^a and the descending B^3^a or B^3^ type (**Table 6**). Moreover, the incidence of AX^3^a was increased in patients with the descending B^3^a or B^3^ type (P < 0.005). This can be clarified by the paralleling correlation between pulmonary segmental arteries and pulmonary segmental bronchi. Thus, when the AX^3^a is identified preoperatively, the thoracic surgeons must investigate the possibility of the descending B^3^a or B^3^. For B^1 + 2^, B^3^a, B^3^b+c type, when S^3^a segmentectomy was planned and performed, it was practical and safe to ligate the AX^3^a originating from PI in the side of the oblique fissure, followed by the B^3^a (**Figure 4**). For B^1 + 2^a+b, B^1 + 2^c+B^3^a, B^3^b+c type, it was easier to dissect the B^3^a from the oblique fissure in the S^3^a segmentectomy (**Figure 4**). And AX^3^a originating MLA was identified after resection B^3^a. At the same time, it may be disturbed by the LUL vein.

It is significant to understand the branching patterns of A^1 + 2^ pre-operatively (**Figure 5**; **Table 7**). When A^1 + 2^ originated from the posterolateral portion of LPA (**Figure 5A**), during S^1 + 2^a segmentectomy, it is necessary to dissect the branches of A^1 + 2^ in a center-to-periphery direction to distinguish A^1 + 2^a, A^1 + 2^b, and A^1 + 2^c. However, it significantly increased the difficulty of dissection. When A^1 + 2^a+b originated from the posterolateral portion of LPA and A^1 + 2^c originated from the interlobar portion of LPA (type H), during S^1 + 2^ segmentectomy, we need to dissect the posterolateral portion and the interlobar portion of LPA to discriminate A^1 + 2^a+b and A^1 + 2^c (**Figure 5A**).

As shown in **Figure 5** and **Table 7**, the branching patterns of the A^1 + 2^ in this study were somewhat different from the previous report (12). The main reason is the introduction of AX^1 + 2^c. In the present study, AX^1 + 2^c arose from the PI in 74 cases (13.7%) (**Figure 2B**). This compares with the figures revealed by Deng (6.8%) and Murota (3.8%) (9, 11). Moreover, AX^1 + 2^c originating from A^3^ was observed in 28 patients (5.2%) and was the first reported (**Figure 2B**).

AX^1 + 2^c is not uncommon in clinical practice; however, if it occurs, it can lead to significant difficulties for patients to perform S^1 + 2^c segmentectomy. No previous report has reported the distribution of AX^1 + 2^c branching patterns in the bronchus type (**Table 8**). Moreover, the combination of branching patterns of the AX^1 + 2^c and the descending B^1 + 2^c type showed significant dependence (**Table 9**). And it was first reported. This denoted that the AX^1 + 2^c was often combined with the descending B^1 + 2^c type. The paralleling relationship between pulmonary segmental arteries and pulmonary segmental bronchi may explain this phenomenon. Thus, when the AX^1 + 2^c was recognized preoperatively, it is crucial to keep an eye on the existence of the descending B^1 + 2^c type. For B^1 + 2^a+b, B^1 + 2^c, B^3^ type, when S^1 + 2^c segmentectomy was implemented, it was feasible and secured to dissect the AX^1 + 2^c originating from PI in the side of the oblique fissure, followed by the B^1 + 2^c (**Figure 6**). When AX^1 + 2^c arose from A^3^, it ran deep within the lung parenchyma of S^3^. For B^1 + 2^a+b, B^3^+B^1 + 2^c type, it was easier to identify the B^1 + 2^c from the oblique fissure in the S^1 + 2^c segmentectomy (**Figure 6**). However, the AX^1 + 2^c was exposed by lifting the B^1 + 2^c stump. And it was essential to avoid causing damage to the A^3^ when the AX^1 + 2^c was ligated. In sum, we defined the coexistence of the AX^1 + 2^c and the descending B^1 + 2^c, the AX^3^a and the descending B^3^a, and the AX^3^a and the descending B^3^ as “Hebei’s triad combinations”.

## Conclusions

This is the first report to explore the associated pulmonary anatomical features of AX^1 + 2^c and AX^3^a. We found that the incidence of AX^1 + 2^c was increased in patients with the descending B^1 + 2^c type, and AX^3^a was increased in patients with the descending B^3^a or B^3^ type. This knowledge will assist in the preoperative planning of S^1 + 2^c segmentectomy and S^3^a segmentectomy.

## Data availability statement

The original contributions presented in the study are included in the article/supplementary material. Further inquiries can be directed to the corresponding author.

## Ethics statement

The Research Ethics Committee approved this retrospective study at Hebei General Hospital (no. 2022119). The need for patient consent was waived because of the retrospective nature of the study.

## Author contributions

ZL: project design and initiation, data analysis, manuscript writing. QZ: project design and initiation, data analysis, manuscript writing. WW: project design and initiation, data analysis, manuscript writing. ZH: data collection. XZ: supervisor. All authors contributed to the article and approved the submitted version.
